# Four Chromosomal Type IV Secretion Systems in *Helicobacter pylori:* Composition, Structure and Function

**DOI:** 10.3389/fmicb.2020.01592

**Published:** 2020-07-10

**Authors:** Wolfgang Fischer, Nicole Tegtmeyer, Kerstin Stingl, Steffen Backert

**Affiliations:** ^1^Max von Pettenkofer-Institut für Hygiene und Medizinische Mikrobiologie, Medizinische Fakultät, LMU München, Munich, Germany; ^2^Department Biologie, Friedrich-Alexander-Universität Erlangen-Nürnberg, Erlangen, Germany; ^3^Department of Biological Safety, National Reference Laboratory for Campylobacter, German Federal Institute for Risk Assessment (BfR), Berlin, Germany

**Keywords:** competence, conjugation, cag, DNA transfer, pathogenicity island, recombination, type IV secretion system, virulence

## Abstract

The pathogenic bacterium *Helicobacter pylori* is genetically highly diverse and a major risk factor for the development of peptic ulcer disease and gastric adenocarcinoma in humans. During evolution, *H. pylori* has acquired multiple type IV secretion systems (T4SSs), and then adapted for various purposes. These T4SSs represent remarkable molecular transporter machines, often associated with an extracellular pilus structure present in many bacteria, which are commonly composed of multiple structural proteins spanning the inner and outer membranes. By definition, these T4SSs exhibit central functions mediated through the contact-dependent conjugative transfer of mobile DNA elements, the contact-independent release and uptake of DNA into and from the extracellular environment as well as the secretion of effector proteins in mammalian host target cells. In recent years, numerous features on the molecular functionality of these T4SSs were disclosed. *H. pylori* encodes up to four T4SSs on its chromosome, namely the Cag T4SS present in the *cag* pathogenicity island (*cag*PAI), the ComB system, as well as the Tfs3 and Tfs4 T4SSs, some of which exhibit unique T4SS functions. The Cag T4SS facilitates the delivery of the CagA effector protein and pro-inflammatory signal transduction through translocated ADP-heptose and chromosomal DNA, while various structural pilus proteins can target host cell receptors such as integrins or TLR5. The ComB apparatus mediates the import of free DNA from the extracellular milieu, whereas Tfs3 may accomplish the secretion or translocation of effector protein CtkA. Both Tfs3 and Tfs4 are furthermore presumed to act as conjugative DNA transfer machineries due to the presence of tyrosine recombinases with cognate recognition sequences, conjugational relaxases, and potential origins of transfer (*oriT)* found within the *tfs3* and *tfs4* genome islands. In addition, some extrachromosomal plasmids, transposons and phages have been discovered in multiple *H. pylori* isolates. The genetic exchange mediated by DNA mobilization events of chromosomal genes and plasmids combined with recombination events could account for much of the genetic diversity found in *H. pylori*. In this review, we highlight our current knowledge on the four T4SSs and the involved mechanisms with consequences for *H. pylori* adaptation to the hostile environment in the human stomach.

## Introduction

About half of the human population carries the pathogen *Helicobacter pylori* in their stomachs, which is well-known for its causative association with severe diseases such as peptic ulcers, MALT lymphoma, or gastric adenocarcinoma. The enormous global prevalence of infection with these bacteria, as well as their classification as an important group I carcinogen (Hooi et al., 2017; de Martel et al., 2020), highlight the importance of *H. pylori* as a major human pathogen. Furthermore, increasing antibiotic resistance rates have led to its inclusion in a high-priority group of pathogens for which research and development of new antibiotics is advisable (Tacconelli et al., 2018).

Since the bacteria are usually acquired in early childhood and persist for decades if untreated, *H. pylori* typically causes chronic infections (Salama et al., 2013). As a consequence, the bacteria have spread with human populations over at least 100,000 years, so that there is a long history of co-evolution with its host (Moodley et al., 2012). Furthermore, individual clinical isolates display an exceptional genetic diversity resulting both from frequent mutation and recombination events, facilitating host adaptation (Suerbaum and Josenhans, 2007). However, genetic diversity is not only achieved by mutation and genetic drift, but also the result of horizontal gene transfer events, which can be considered an important feature of the lifestyle of *H. pylori*. Specifically, *H. pylori* is naturally competent for transformation and highly recombination-proficient, so that exchange of chromosomal DNA fragments between strains is frequent and highly efficient. Moreover, classical mobile genetic elements, such as plasmids (Höfler et al., 2004), phages (Vale and Lehours, 2018), transposable elements (Kersulyte et al., 1998; He et al., 2015), or genome islands, are present in the *H. pylori* population as well.

**FIGURE 1:**
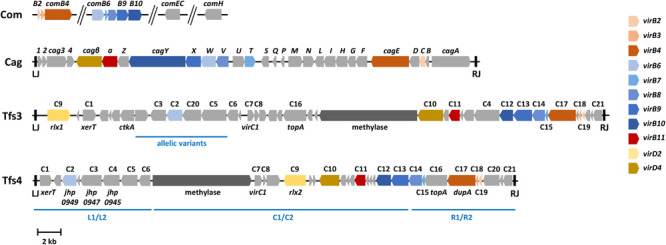
Genetic arrangements of four chromosomally encoded type IV secretion system gene clusters in *H. pylori*. The arrows show typical arrangements of the *cag*, *comB*, *tfs3*, and *tfs4* genes, with coloring according to proven T4SS functions and/or to sequence similarities with prototypical *virB*/*virD* genes, as indicated. The Cag, Tfs3, and Tfs4 systems are located on genome islands delimited by left (LJ) and right junctions (RJ), which consist of 31 bp (Cag) or 7 bp (Tfs3, Tfs4) direct sequence repeats. The competence (*com*) genes are not organized in a contiguous unit, but comprise two operons (*comB2* to *comB4* and *comB6* to *comB10*) with genes homologous to the corresponding *virB* genes, and the *comEC* and *comH* genes located elsewhere in the genome. Gene designations for *cag* pathogenicity island genes represent the commonly used names, whereas genes conserved between ICE*Hptfs3* and ICE*Hptfs4* (C1 to C21) are indicated, together with some additional or alternative designations, according to Delahay et al. (2018), to illustrate similarities between these two islands. Note that individual genes (e.g., the methylase or *ctkA* genes) may be absent from some ICEs, and that most ICE*Hptfs4* genes and some ICE*Hptfs3* genes (e.g., C2, C3, C5) may occur in distinct allelic variants (regions where such variants occur are indicated by blue bars). On ICE*Hptfs4* islands, variants are usually grouped as left (L1/L2), central (C1/C2) or right (R1/R2) regions, which can be combined to form distinct ICE variants, such as L1C1R1, L2C1R2, etc.

Three genome islands, all of which encode complete or partial T4SSs, have been described in *H. pylori*. The 40 kb *cag* pathogenicity island is integrated at a specific genome locus and flanked by direct 31 bp repeats (Censini et al., 1996), but may be rearranged in some strains (Fischer, 2011; Su et al., 2019). It encodes the Cag T4SS and its effector protein, the cytotoxin-associated antigen CagA, both of which are well-known as major *H. pylori* virulence factors. Two further genome islands, which can be variably integrated in different genome positions, typically producing 7 bp direct repeats, are now designated ICE*Hptfs3* and ICE*Hptfs4* and encode the Tfs3 and Tfs4 T4SSs, respectively, (Figure 1). Genetic and functional data indicate that these two genome islands actually represent integrating conjugative elements (ICEs), with the implication that the Tfs3 and Tfs4 apparatuses are conjugation systems involved in horizontal gene transfer (Fischer et al., 2014). While these three T4SSs are located on genomic islands and are thus variably present, *H. pylori* also contains a fourth T4SS within its core genome, the ComB DNA uptake system (Hofreuter et al., 2001). That *H. pylori* employs a T4SS for DNA uptake, while most other naturally competent bacteria use other machineries for the same purpose, highlights a general preference of these bacteria for T4SSs. Furthermore, the fact that individual strains may thus contain up to four different T4SSs with at least partially overlapping functional modules, raises important questions with respect to substrate specificity and strain evolution. This review discusses recent studies related to the structure and function of all four T4SSs in *H. pylori*, but also to evolutionary aspects associated with these systems.

## The T4SS Encoded by the *Cag* Pathogenicity Island

**FIGURE 2:**
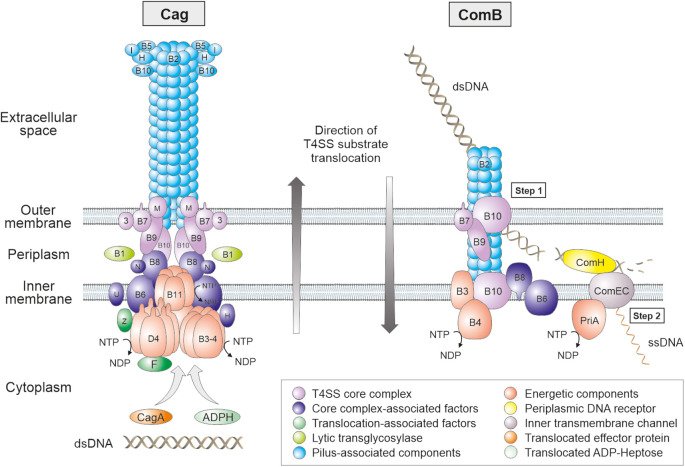
Models for the assembly and subcellular localization of the Cag and ComB type IV secretion systems in the membranes of *H. pylori*. Proteins sharing sequence homology to the *Agrobacterium tumefaciens* VirB/VirD4 T4SS are marked with B1 to B11 or D4, respectively. Accessory Cag and Com proteins are designated with letters as given in Figure 1. The Cag T4SS pilus proteins, core complex components, NTP-hydrolysing energetic factors, translocated effector molecules (CagA, ADP-Heptose and dsDNA) and other proteins are emphasized with different colors as denoted in the legend at the bottom. The proposed position of the T4SS proteins is presented in a simplified fashion. In the ComB T4SS, an outer membrane complex might be composed of ComB7, B9 and B10. ComB3, B4, B6, B8 and B10 form a complex at the inner membrane. Hypothetically, the pilin subunit ComB2 binds to dsDNA at the surface of the bacterium (pseudopilus). By retraction of these pilin subunits, powered by the ATPase ComB4, bound dsDNA is translocated into the periplasm. ComH guides incoming dsDNA to the ComEC channel by binding dsDNA with its C-terminus and docking its N-terminus to a periplasmic domain of ComEC. In the cytoplasm, the helicase PriA might empower uptake of ssDNA. Please note that effector molecule delivery in both T4SSs proceeds in opposite directions as indicated. In contrast, almost nothing is known about the assembly and subcellular localization of Tfs3 and Tfs4 components.

The *cag*PAI, which encodes the first and best characterized T4SS in *H. pylori*, comprises a locus of approximately 40 kb carrying about 28 genes (Figure 1). This T4SS is present in highly virulent *H. pylori* strains and absent in less virulent isolates. Thus, the *cag*PAI has been described as a genetic marker for gastric disease development (Covacci and Rappuoli, 2000; Yamaoka, 2008; Salama et al., 2013). The transporter encoded on the *cag*PAI comprises all VirB1 to VirB11 and VirD4 orthologs found in the prototypical T4SS of *Agrobacterium tumefaciens* (Backert et al., 2017). However, assembling of the *cag*PAI machinery depends on a dozen other Cag proteins, making this T4SS unique (Fischer et al., 2001; Backert et al., 2015; Figure 2). Several recent investigations have visualized T4SS structures in *H. pylori* cells, or isolated T4SS subcomplexes, using single-particle electron microscopy or cryo-electron tomography techniques (Frick-Cheng et al., 2016; Chang et al., 2018, Chung et al., 2019; Hu et al., 2019). Imaging the T4SS core structure by electron microscopy revealed some structural similarity with the T4SS core complex “VirB3-10” encoded by the conjugative *Escherichia coli* plasmid R388 (Low et al., 2014; Frick-Cheng et al., 2016). Nevertheless, the *cag*PAI subassembly is substantially bigger with a diameter of 41 nm compared to 28 nm for R388. In addition, mass spectrometry revealed that the Cag T4SS core structure comprises five proteins, i.e., CagT (similar to VirB7), CagX (VirB9), CagY (VirB10), CagM and Cag3, versus three proteins in R388 (VirB7, VirB9, VirB10) (Low et al., 2014; Frick-Cheng et al., 2016; Chung et al., 2019). Thus, *H. pylori* exhibits two additional T4SS core subunits (CagM and Cag3). This core complex is supposed to be connected to an extracellular T4SS pilus, whose composition and assembly are still not well-understood (Backert et al., 2015). A unique protein in the Cag T4SS is CagY, which is related to VirB10 of the T4SSs in other bacteria. However, CagY exhibits an additional large N-terminal domain containing two large repeat segments, providing an extraordinary structural variability in CagY both by in-frame deletion or duplication events (Delahay et al., 2008; Barrozo et al., 2013). Remarkably, these rearrangements in CagY, which were sufficient to trigger loss or gain of T4SS functions, are driven by the host immune response (Barrozo et al., 2013), or can be induced by the cancer chemopreventive agent α-difluoromethylornithine (Sierra et al., 2019). Consequently, it was proposed that CagY may operate as a molecular “switch” or a “rheostat” to fine tune host inflammatory responses in order to sustain persistent *H. pylori* infection. Interestingly, electron microscopy further showed that T4SS pilus formation upon infection in the polarized gastric epithelium appears primarily at basolateral surfaces, but not at apical membranes (Tegtmeyer et al., 2017b). To achieve this goal, it appears that *H. pylori* secretes a Cag T4SS-independent factor, the serine protease HtrA, into the supernatant by a yet unknown mechanism, which cleaves the surface-associated junctional proteins claudin-8, occludin and E-cadherin, followed by opening of tight and adherens junctions and paracellular transmigration of *H. pylori* (Schmidt et al., 2016; Tegtmeyer et al., 2017b). In this way, the bacteria can travel across the host epithelial monolayer and inject CagA at basolateral sites.

**FIGURE 3:**
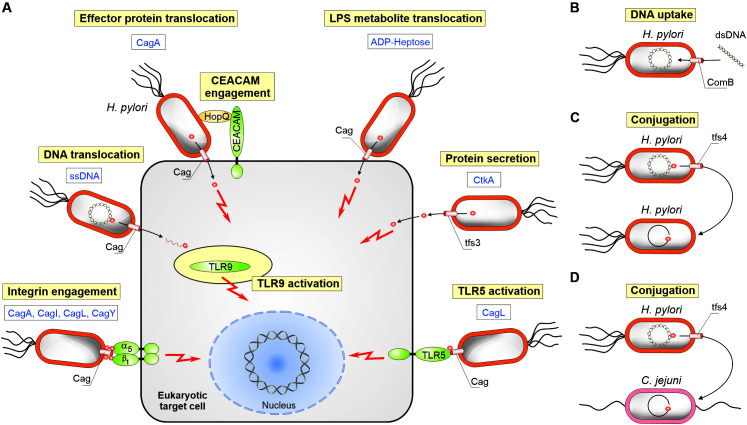
Schematic presentation for the functional importance of four chromosomally encoded type IV secretion systems of *H. pylori.* **(A)** Effector molecule translocation into human host target cells comprises CagA, CtkA, ADP-heptose, and chromosomal DNA. The indicated T4SSs of the *cag*PAI and ICE*Hptfs3* are involved. Delivery of CagA requires the interaction of adhesin HopQ with CEACAM receptors. In addition, several indicated pilus-associated *cag*PAI proteins can interact directly with other cell surface receptors (integrins and TLR5) to induce signaling. **(B)** The ComB system functions to deliver naked DNA from the environment into *H. pylori*. Conjugative DNA transfer has been also observed among *H. pylori* strains **(C)**, or from *H. pylori* to other bacteria such as *Campylobacter jejuni* **(D)**.

The Cag T4SS is not only remarkable with regard to its size and composition, but also regarding the variety of transported molecules, which include protein, LPS metabolite and chromosomal DNA substrates to be delivered into gastric epithelial cells (Figure 3A). The only known translocated effector protein of the T4SS is CagA (Covacci and Rappuoli, 2000). CagA was originally discovered as a highly immunogenic antigen in patients, is variable in size (about 120–140 kDa) and has no homology to any other known protein (Backert et al., 2017). Previous studies disclosed numerous 3D structures of the 100 kDa amino-terminal domain of CagA (Hayashi et al., 2012; Kaplan-Türköz et al., 2012). Following injection into cultured gastric epithelial cells, or gastroids (Boccellato et al., 2019), CagA becomes tyrosine-phosphorylated by host cell kinases of the Src and Abl families (Mueller et al., 2012). Subsequently, intracellular CagA was shown to interact with at least 25 signaling factors such as SHP2, Crk, Par1 or phosphatidyl-inositol-3-kinase (Higashi et al., 2002; Suzuki et al., 2005; Saadat et al., 2007; Selbach et al., 2009; Zhang et al., 2015). In this way, translocated CagA hijacks numerous elementary host signal transduction pathways including cell polarity, adhesion, proliferation and anti-apoptosis (Naumann et al., 2017; Tegtmeyer et al., 2017a). In addition, infection studies and other functional analyses in Mongolian gerbils (Franco et al., 2008), transgenic mice (Ohnishi et al., 2008), *Drosophila* (Reid et al., 2012), zebrafish (Neal et al., 2013), as well as stem cells in humans and mice (Sigal et al., 2015) have demonstrated that *cagA* expression is required and sufficient to trigger cell proliferation and even malignancy. Moreover, the Cag T4SS was described to trigger a strong pro-inflammatory response in gastric epithelial cells (Backert and Naumann, 2010). It was reported that delivered chromosomal DNA (Varga et al., 2016), and LPS biosynthesis intermediates (Gall et al., 2017; Stein et al., 2017; Zimmermann et al., 2017), most importantly ADP-glycero-β-D-manno-heptose (ADP-heptose) (Pfannkuch et al., 2019), play roles in this scenario. It was shown that a functional *cag*PAI T4SS is required for activation of toll-like receptor-9 (TLR9), and that chromosomal DNA is actively delivered to employ this innate immune receptor (Varga et al., 2016). On the other hand, translocated ADP-heptose strongly activates the transcription factor NF-κB pathway through the α-protein kinase 1 (ALPK1)/TRAF-interacting protein with forkhead-associated domain (TIFA) pathway triggering major pro-inflammatory responses by *H. pylori* (Pfannkuch et al., 2019).

Since the discovery of its secretion in 1999, it was assumed for a long time that CagA may be randomly translocated into various host cell types. Interestingly, this is not the case because recent reports provided evidence that various host cell receptors could affect T4SS functions. In this regard, a further difference compared to most other T4SSs is that, besides the canonical VirB2 (CagC) and VirB5 (CagL) orthologs, several additional factors (such as CagA, CagI, and CagY) are exposed at the surface of the Cag T4SS pilus. CagL, and the latter three proteins were all reported to directly interact with the host receptor integrin-α_5_β_1_ with very high affinity (Kwok et al., 2007; Jiménez-Soto et al., 2009; Bönig et al., 2016; Koelblen et al., 2017). However, very recent studies using CRISPR-Cas9 knockout in AGS and Kato-III cells suggest that integrins are not required for the injection of CagA (Zhao et al., 2018), and may have a different function by enhancing integrin-based binding to the extracellular matrix to avoid excessive epithelial cell lifting during infection (Tegtmeyer et al., 2011). Instead, *H. pylori* exploits the carcinoembryonic antigen-related cell adhesion molecule (CEACAM) receptors, via the surface-exposed outer-membrane protein HopQ, for bacterial adhesion and T4SS-dependent delivery of CagA (Javaheri et al., 2016; Königer et al., 2016; Behrens et al., 2020). Remarkably, the HopQ-CEACAM interaction is required for full T4SS function, gastric colonization and pathology, but the exact molecular mechanisms are yet unclear and need to be identified in future studies. Finally, very recent studies have shown that T4SS pilus-associated CagL can act independently of effector molecule translocation as a flagellin-independent activator of toll-like receptor-5 (TLR5) (Pachathundikandi et al., 2019). CagL contains a flagellin D1 domain-like motif that mediates binding to TLR5-positive epithelial cells, TLR5 activation, and downstream signaling *in vitro*. Using Tlr5-knockout and wild-type mice, it was demonstrated that TLR5 expression is required for efficient control of *H. pylori* infection. These results indicate that CagL, by activating TLR5, may modulate Th1 immunity and other immune responses to *H. pylori*.

Besides the function of the Cag T4SS during *H. pylori* infection of gastric epithelial cells, interactions with immune cells were also reported (Blaser et al., 2019). For example, upon T4SS-dependent delivery into various immune cell lines, CagA can be processed by a yet unknown protease into two fragments, with unclear functional outcome (Moese et al., 2001; Odenbreit et al., 2001; Busch et al., 2015). Translocation and phosphorylation of CagA resulted in expression of the heme oxygenase-1 gene in RAW264.7 mouse macrophages (Gobert et al., 2014). Increased levels of heme oxygenase-1 were also found in gastric mononuclear cells from mice and humans infected with CagA-positive strains, and could be correlated with reduced inflammation, and increased bacterial colonization. Macrophage-like cells formed large homotypic aggregates after upregulation of intercellular adhesion molecule 1 (ICAM1), which was dependent on the Cag T4SS, but independent of CagA, and may regulate cell-cell interactions and inflammatory signaling (Moese et al., 2002). In another study, it was shown that the Cag T4SS upregulated the microRNA miR-155 in primary murine bone marrow-derived macrophages (BMMs), while CagA was again not involved (Koch et al., 2012). Microarray experiments revealed several pro-apoptotic genes as targets of miR-155, and miR-155^–/–^ knockout BMMs infected with *H. pylori* were significantly more susceptible to apoptosis compared to wild-type BMMs, suggesting a protective role of miR-155 against DNA damage induced by *H. pylori* infection (Koch et al., 2012). Further studies identified a class of early response genes with low mRNA stability (due to the presence of AU-rich elements in their 3′-UTRs) in infected BMMs, which were expressed earlier upon contact with Cag T4SS-positive than with T4SS-negative strains. This pattern was only seen in the absence of TLR signaling (in MyD88/Trif^–/–^ knockout BMMs), and included the upregulated cytokines TNF-α and IL-1β (Koch et al., 2016). Taken together, more work is required to understand the role of T4SS-mediated immune cell control by *H. pylori*. In this regard, a novel gastric spheroid co-culture model of gastric epithelial cells and immune cells was developed, and seems well suited for immuno-surveillance studies during *H. pylori* infection (Sebrell et al., 2019).

## The Comb T4SS System of *H. pylori*

Natural competence in bacteria is commonly mediated by so-called type IV pili or type IV pilin-like proteins, but the corresponding genes are absent in *H. pylori* (Tomb et al., 1997). Instead, *H. pylori* is unique in using a T4SS named ComB system for DNA import during natural transformation (Figure 3B). The associated genes are clustered in two operons, *comB2*-*comB4* and *comB6*-*comB10* (Hofreuter et al., 2001; Karnholz et al., 2006). Compared to the homologous VirB/VirD4 T4SS of *Agrobacterium*, several key components are missing, such as a VirB1, VirB5, VirD4, and VirB11 homolog (Figures 1, 2). Thus, ComB4 is the only proposed ATPase involved in the ComB system. Structural analysis suggested that ComB6-B10 form a large complex spanning the periplasm from the inner to the outer membrane (Hofreuter et al., 2003; Karnholz et al., 2006). While ComB6 and ComB8 are probably located in the inner membrane, the outer membrane complex might be composed of ComB7, ComB9 and ComB10, according to the crystal structure of the homologous VirB/VirD4 system, forming a central cavity of 3.2 nm (Chandran et al., 2009).

The ComB system functions independent of the Cag T4SS in *H. pylori* (Hofreuter et al., 2001), and is responsible for the first DNA uptake step into the periplasmic space (Stingl et al., 2010; Figure 2). Hence, the system is able to transport double-stranded (ds) DNA through the outer membrane, since the process can be visualized by using fluorescent dyes intercalating into dsDNA. It is, however, unknown whether single-stranded (ss) DNA can also serve as a substrate for the ComB system, as observed for the analogous type IV pilus system in *Neisseria* (Hepp and Maier, 2016). The second step of DNA transport over the inner membrane is mediated by the channel formed by ComEC, which transports ssDNA into the cytoplasm (Yeh et al., 2003; Stingl et al., 2010). Laser tweezer analysis showed that dsDNA is imported at high velocity of 1.3 kbp/s, comparable to pilus retraction processes (Stingl et al., 2010). Moreover, the capacity of DNA import is substantial, with median rates of 350 kb within 10 min, and up to one chromosomal equivalent per cell (Krüger et al., 2016). It appears that ComB transports dsDNA of any source. By comparison, in the analogous systems using type II secretion/type IV pilus proteins, DNA uptake velocities are much lower (Hepp and Maier, 2016), and the total capacity is limited to around 40 kbp (Gangel et al., 2014). The driving force for pulling the DNA over the outer membrane in these systems is generated by non-specific binding of DNA to the periplasmic receptor protein ComE (Gangel et al., 2014; Seitz et al., 2014), but a homolog of ComE was not identified in *H. pylori*.

However, the unique ComH protein was shown to be located in the periplasm and to bind incoming DNA in *H. pylori* (Damke et al., 2019). The C-terminal domain of ComH is essential and sufficient for uptake into the periplasm, while the N-terminus was demonstrated to interact with a periplasmic domain of the inner membrane channel ComEC, suggesting that ComH delivers incoming DNA to be further transported into the cytoplasm. Periplasmic co-localization of imported DNA and ComH was maximal after 90 min, while DNA uptake into the periplasm was completed within much shorter times (∼10 min), indicating that ComH might not be directly involved in generating the pulling force for DNA uptake across the outer membrane. More likely, ComH is important for transfer of periplasmic DNA from ComB to the membrane channel ComEC (Figure 2). The pilin VirB2 was shown to directly interact with DNA in the agrobacterial T4SS (Cascales and Christie, 2004). Thus, it remains to be investigated in future studies, if ComB2 has a similar role in the ComB-dependent DNA uptake system before periplasmic DNA interacts with ComH to be introduced into the ComEC channel for further uptake into the cytoplasm.

There is evidence that ComB-dependent DNA uptake is not a constitutive process, but underlies strong regulation (Krüger et al., 2016). The competent state does not lead to growth arrest as observed in other bacteria (Corbinais et al., 2016). The major signal for induction of the system is a neutral pH > 6.5, and ComB activity is switched off at slightly acidic pH *in vitro*, suggesting that DNA uptake occurs in close contact to gastric epithelial cells during infection *in vivo* (Krüger et al., 2016). Moreover, it was shown that oxidative stress modulates competence development (Krüger et al., 2016). Natural transformation activity might be mainly regulated at the level of outer membrane transport. The amounts of ComB8 and ComB10 proteins correlated with the activity of outer membrane transport and transformation rates, and overexpression of *comB6-B10* enhanced competence (Corbinais et al., 2017). Overall, it was suggested that the uptake of vast amounts of DNA by this highly efficient T4SS serves for enhanced genetic diversity. Moreover, it might also play a role in oxidative stress defense by protecting the chromosome via generation of a large periplasmic pool of foreign DNA. Infection studies in mice and Mongolian gerbils using *H. pylori comB10* and/or *dprA* mutants indicated that natural transformation might convey a benefit for long-term rather than initial colonization (Kavermann et al., 2003; Dorer et al., 2013). However, systematic *in vivo* studies are required in future in order to decipher the impact of natural transformation for chronic persistence and bacterial dissemination.

## Tfs3 and Secretion of Cell Translocating Kinase a (CtkA)

Genes belonging to the additional Tfs3 and Tfs4 T4SSs were originally discovered in a region of high genome plasticity observed among the first two sequenced *H. pylori* strains, 26695 and J99 (Kersulyte et al., 2003). After comparative analysis of more *H. pylori* genome sequences, it became evident that these T4SS genes occur in different variants that were either considered as subtypes *tfs3*, *tfs3a*, and *tfs3b* (Kersulyte et al., 2009), or as separate systems termed *tfs3* and *tfs4* (Fischer et al., 2010). The latter designations were chosen to point out that these genes are actually not more closely related to each other than to the *comB* genes, and they are also used here. Furthermore, it became clear that *tfs3* and *tfs4* genes are not restricted to the original “plasticity zones”, but can alternatively be found in many other genomic locations, where they are organized together with further genes as genome islands (Figure 1; Kersulyte et al., 2009; Fischer et al., 2010, 2014). Typical features in addition to the T4SS genes, such as flanking sequence duplications (5′-AAGAATG-3′), the presence of *xer* recombinase and *rlx* relaxase genes, and potential *oriT* sequences (Grove et al., 2013), indicated that these islands represent integrating conjugative elements (ICEs), termed ICE*Hptfs3* and ICE*Hptfs4* (Fischer et al., 2014).

Full-length ICE*Hptfs3* elements, as well as versions with truncated *tfs3* genes, or lacking several genes, have been detected in numerous clinical *H. pylori* isolates (Kersulyte et al., 2003; Alvi et al., 2007; Fischer et al., 2014; Gong et al., 2015; Romo-González et al., 2015; Delahay et al., 2018). A possible role of Tfs3 has been suggested in DNA transfer (Kersulyte et al., 2003), supported by the finding that the Tfs3 relaxase (Rlx1) is involved in mobilization of a plasmid with an origin of transfer from plasmid RP4 (Backert et al., 2005), but the actual function of Tfs3 still remains widely unclear. However, the serine/threonine kinase CtkA (for cell translocating kinase A, corresponding to gene *jhp940*) was discovered to be present, but only in a subset of ICE*Hptfs3*-positive strains. The crystal structure of CtkA has been resolved, which revealed that JHP940 is the first example of a eukaryotic-like serine/threonine kinase in *H. pylori* (Kim et al., 2010). It was further shown that purified GFP-tagged CtkA is taken up by cultured human (HeLa) cells by a yet unknown mechanism. In addition, transient transfection of AGS cells revealed that CtkA is translocated from the cytosol into the nucleus in a time-dependent manner. Functionally, it was found that CtkA can indirectly up-regulate the phosphorylation and activation of NF-κB subunit p65 at serine residue 276 through a yet unknown pathway (Kim et al., 2010). In addition, recombinant CtkA was observed to diminish the survival of RAW264.7 mouse macrophage cells by about 55% within 24 h of infection (Tenguria et al., 2014). This decreased cellular viability was due to cell apoptosis, and involved caspase-1, which was activated by treatment of cells with purified CtkA. Further *in vitro* studies demonstrated that CtkA could also act as an auto-phosphorylating tyrosine kinase and induce the dose- and time-dependent secretion of pro-inflammatory cytokines IL-1β, TNF and IL-6 in RAW264.7 cells (Tenguria et al., 2014). Together, these data suggested that CtkA presumably represents an ICE*Hptfs3*-encoded pro-inflammatory and pro-apoptotic regulator, which can kill macrophages (Tenguria et al., 2014). In a more recent study, CtkA interaction with AGS gastric epithelial cells was discovered to be dependent upon Tfs3 secretion apparatus genes, but independent of the Tfs4 or Cag T4SSs (Alandiyjany et al., 2017). Together, these observations identified CtkA as a putative secreted Tfs3 T4SS effector protein (Figure 3A), and suggested a role for the Tfs3 T4SS in CtkA-mediated translocation and pro-inflammatory signal transduction by *H. pylori* (Alandiyjany et al., 2017). Thus, the *tfs3* gene cluster and CtkA may represent novel *H. pylori* virulence factors with a possible role in inducing chronic inflammation to ensure bacterial survival and persistence. However, the actual transport of CtkA by the Tfs3 T4SS is not yet fully clear. CtkA can be found secreted in the supernatant of cultured *H. pylori* (Tenguria et al., 2014), suggesting some similarity to secreted T4SS effectors like *Bordetella pertussis* toxin (Grohmann et al., 2018), but it remains to be studied how CtkA can be taken up across the host cell membrane or if it can also be directly injected into the host cell. It would be also interesting to investigate if Tfs3 is functional for other T4SS substrate molecules.

## The Tfs4 Secretion System and Ice Physiology

Similar to ICE*Hptfs3* elements and their cognate Tfs3 secretion systems, ICE*Hptfs4* islands harbor genes for all known T4SS functions, and further genes were predicted to encode important functions for ICE physiology, such as *xerT* recombinase, topoisomerase, DNA methylase, or *virC1* (*parA*) relaxosome assembly genes (Figure 1). Moreover, several additional genes with unknown functions also have counterparts with moderate sequence similarities on the ICE*Hptfs3* elements (Delahay et al., 2018), but are partly organized in different putative transcriptional units, indicating that both ICE types have analogous basic functions. Some further genes are specific for ICE*Hptfs4* and might have maintenance functions, for example as toxin-antitoxin systems, or might represent accessory genes that possibly encode effector proteins (similar to *ctkA*, which occurs uniquely on ICE*Hptfs3* elements). However, there is currently no direct evidence for the function of any of these accessory ICE*Hptfs4* genes. Somewhat different from ICE*Hptfs3* elements, where only few genes are variable, almost all ICE*Hptfs4* genes occur as one of two distinct alleles. Individual alleles are linked to form modules in the left, central, or right parts of the island, respectively, which can be combined to form different ICE variants (Delahay et al., 2018; Figure 1). Hybrid ICE*Hptfs3*/ICE*Hptfs4* arrangements may also occur, mostly due to recombination events between the highly homologous methylase genes (Fischer et al., 2014).

Despite the absence of functional data for most ICE*Hptfs4* genes, several genes have been correlated with disease outcome. The most prominent of these disease-associated genes is the *dupA* (duodenal ulcer-promoting) gene, which is actually the *virB4* homolog of one ICE*Hptfs4* right module (Figure 1). This gene was originally associated with duodenal ulcer risk in East Asian and South American populations (Lu et al., 2005), but later studies have shown that disease association might be population-specific (Shiota et al., 2010), and that the presence of further genes in addition to *dupA* is generally a better predictor of disease outcome (Jung et al., 2012). The latter observation suggested that not the *dupA* gene as such, but rather the whole Tfs4 T4SS, and possibly its secreted effector molecules, are the actual drivers of disease development. Further ICE*Hptfs4* genes (*jhp0945*, *jhp0947*, *jhp0949*; Figure 1) have indeed been correlated with disease as well [reviewed in Waskito et al. (2018)], but their functional role within the Tfs4 system and/or in interaction with host cells remain to be identified.

Typical ICEs encode recombinase functions that excise the elements from their host chromosome to form circular intermediates, which may subsequently be transferred by conjugation to recipient cells (Wozniak and Waldor, 2010). Consistent with this concept, ICE*Hptfs4* has been shown to form such circular products, depending on the putative site-specific recombinase XerT, the presence of the (duplicated) 5′-AAGAATG-3′ integration motif, and the *xerT* upstream region containing a circularization-dependent promoter (Fischer et al., 2010; Weiss et al., 2019). The reported nicking activity of the Tfs4 relaxase (Rlx2) with a recognition site upstream of its own gene, and its interaction with the putative Tfs4 relaxosome protein VirC1 (Grove et al., 2013), strongly suggest that ICE*Hptfs4* can be transferred in the canonical way (Figure 3C), and that Rlx2 is thus a Tfs4-secreted protein. It has indeed been demonstrated that ICE*Hptfs4* can be transferred horizontally in the presence of extracellular DNases, indicating such a conjugative transfer process (Fischer et al., 2010). However, a recent study showed that prior excision of ICE*Hptfs4* from the chromosome via the AAGAATG motifs, as well as the presence of the T4SS genes, are not absolutely required for transfer (Weiss et al., 2019), indicating other transfer routes. In fact, these observations suggest that the mobilization of chromosomal DNA by yet unknown mechanisms is possible, a conclusion that has also been drawn from the fact that chromosomal markers can be transferred by a conjugative process from *H. pylori* to the related pathogen *Campylobacter jejuni* (Oyarzabal et al., 2007; Figure 3D). Furthermore, they demonstrate that homologous recombination is more efficient in *H. pylori* than site-specific recombination. Nevertheless, they do not exclude that canonical ICE transfer and integration events occur at lower rates, an assumption which is supported by the observed integration of ICE*Hptfs4* (as well as ICE*Hptfs3*) in many different genomic locations (Fischer et al., 2014). Interestingly, neither the Tfs3 nor the Tfs4 systems are required for conjugative transfer of naturally occurring *H. pylori* plasmids, although these plasmids do not contain their own conjugation systems (Rohrer et al., 2012).

ICE*Hptfs4* elements are found in all *H. pylori* populations, including the *cag*PAI-negative hpAfrica2 population (Delahay et al., 2018). Furthermore, the presence of ICE genes (Gressmann et al., 2005), and the production of relaxases (Tegtmeyer et al., 2013), has also been documented in the closely related species *Helicobacter acinonychis*, indicating a long-lasting evolutionary association with *H. pylori*. However, an erosion of the T4SS genes by frameshifts or deletions is visible in numerous strains from different populations (Fischer et al., 2014; Delahay et al., 2018), suggesting that evolutionary advantages may be conferred upon the harboring strains by the additional genes rather than the type IV secretion genes themselves. Elucidating individual activities of these additional genes and their contribution to bacterial fitness or host adaptation will thus be important for understanding ICE function.

## Conclusion

*Helicobacter pylori* represents one of the most successful human pathogens (Salama et al., 2013). Two main reasons for this success are its effective virulence factors and the high genetic variability among strains. Genetic diversity is typical for various human pathogens that need to persist in the human host and survive under hostile environmental conditions. The diversity in gene content among *H. pylori* strains stimulated research interest in how DNA is gained and lost during bacterial evolution. It has been considered that T4SSs play an important role in this scenario (Grohmann et al., 2018). One of the molecular mechanisms that *H. pylori* uses to gain exogenous DNA is by natural transformation using the ComB T4SS machinery, and the exchange of DNA by putative conjugative T4SS gene clusters present in the *H. pylori* genome. Furthermore, the increasing number of reports on transferable plasmids (Kleanthous et al., 1991; Hofreuter and Haas, 2002; Höfler et al., 2004; Joo et al., 2012; Rohrer et al., 2012) and phages (Schmid et al., 1990; Heintschel von Heinegg et al., 1993; Lehours et al., 2011; Luo et al., 2012; Uchiyama et al., 2012) in *H. pylori* strains encouraged researchers to propose that DNA transfer events through conjugation and phage transduction would also play an important role in *H. pylori* genetic diversity (Vale and Lehours, 2018; Waskito and Yamaoka, 2019). These discoveries and their role in producing genetic diversity in *H. pylori* should be investigated in more detail in future. In addition, an intriguing possibility is that ssDNA export and integration in the human host cell chromosome could play a role in colonization and disease development by *H. pylori* in the stomach, as compared to the T-DNA transfer by *Agrobacterium* resulting in crown gall tumors in infected plants. There are many other open questions, for example about how the conjugative T4SSs work in detail, and if there are yet unknown translocated effector molecules. For the Cag T4SS, further translocated effector proteins besides CagA, such as CagQ (HP0535) and others, have been predicted (Olbermann et al., 2010), but this awaits functional analysis. Thus, studying novel T4SS functions in *H. pylori* and related bacteria is a rewarding research topic also in future.

## Author Contributions

All authors listed have made a substantial, direct and intellectual contribution to the work, and approved it for publication.

## Conflict of Interest

The authors declare that the research was conducted in the absence of any commercial or financial relationships that could be construed as a potential conflict of interest.
